# Remote Effects of Lower Limb Ischemia-Reperfusion: Impaired Lung, Unchanged Liver, and Stimulated Kidney Oxidative Capacities

**DOI:** 10.1155/2014/392390

**Published:** 2014-08-10

**Authors:** Z. Mansour, A. L. Charles, M. Kindo, J. Pottecher, T. N. Chamaraux-Tran, A. Lejay, J. Zoll, J. P. Mazzucotelli, B. Geny

**Affiliations:** ^1^Equipe d'Accueil 3072 “Mitochondries, Stress Oxydant et Protection Musculaire”, Fédération de Médecine Translationnelle, Institut de Physiologie, Universite de Strasbourg, 67000 Strasbourg, France; ^2^Service de Chirurgie Cardiovasculaire, Pole de Cardiologie, Hopitaux Universitaires, CHRU de Strasbourg, 67000 Strasbourg, France; ^3^Service de Physiologie et d'Explorations Fonctionnelles, Pole de Pathologie Thoracique, Hopitaux Universitaires, CHRU de Strasbourg, 67000 Strasbourg, France

## Abstract

Remote organ impairments are frequent and increase patient morbidity and mortality after lower limb ischemia-reperfusion (IR). We challenged the hypothesis that lower limb IR might also impair lung, renal, and liver mitochondrial respiration. Two-hour tourniquet-induced ischemia was performed on both hindlimbs, followed by a two-hour reperfusion period in C57BL6 mice. Lungs, liver and kidneys maximal mitochondrial respiration (*V*_max⁡_), complexes II, III, and IV activity (*V*_succ_), and complex IV activity (*V*_TMPD_) were analyzed on isolated mitochondria. Lower limb IR decreased significantly lung *V*_max⁡_ (29.4 ± 3.3 versus 24 ± 3.7 *μ*mol O_2_/min/g dry weight, resp.; *P* = 0.042) and tended to reduce *V*_succ_ and *V*_TMPD_. IR did not modify liver but increased kidneys mitochondrial respiration (79.5 ± 19.9 versus 108.6 ± 21.4, *P* = 0.035, and 126 ± 13.4 versus 142.4 ± 10.4 *μ*mol O_2_/min/g dry weight for *V*_max⁡_ and *V*_succ_, resp.). Kidneys mitochondrial coupling was increased after IR (6.5 ± 1.3 versus 8.8 ± 1.1, *P* = 0.008). There were no histological changes in liver and kidneys. Thus, lung mitochondrial dysfunction appears as a new early marker of hindlimb IR injuries in mice. Further studies will be useful to determine whether enhanced kidneys mitochondrial function allows postponing kidney impairment in lower limb IR setting.

## 1. Introduction

Peripheral arterial disease is a frequent pathology and corresponds to a major public health problem. The symptomatology ranges from intermittent claudication to tissue necrosis needing amputation [1]. A better knowledge on ischemia and reperfusion-specific deleterious effects results in new therapies able to reduce skeletal muscle mitochondrial dysfunctions such as ischemic and/or pharmacologic pre- or postconditioning. Thus, reducing oxidative stress and skeletal muscle mitochondrial dysfunction becomes a major goal in the setting of lower limb ischemia-reperfusion (IR) [2, 3].

Importantly, even subtle muscle damage can lead to significant remote organ injuries, and, thus, improving our knowledge on the mechanisms involved in such lesions appears mandatory. Indeed, lower limb ischemia-reperfusion is well known to result in remote organ injuries, key factors increasing perioperative and long-term morbidities [4–8].

Pulmonary injuries have been described after aortic cross-clamping, in humans and in animals, and systemic inflammation has been incriminated along with leukocytes sequestration in lung parenchyma. Histology also showed partial atelectasis with collapsed and pinched alveoli, thicker and felted alveolar walls. Pulmonary damages have been thus largely involved in patient mortality and morbidity through decreased FIO_2_/PaO_2_ ratio and impaired oxygenation [6–12].

Liver damage related to lower limb ischemia-reperfusion was less demonstrated but, in view of the detoxification role of the liver, it might concentrate inflammatory cells triggered by lower limb IR and should be affected, as previously reported in the setting of gut IR [13].

Kidneys are particularly concerned in the remote alteration phenomenon after skeletal muscle ischemia-reperfusion injuries. Yassin et al. showed renal dysfunction on a Wistar rat model with bilateral hindlimb IR injuries [8]. Gyurkovics et al. described histological lesions with swollen, vacuolated tubular cells and precipitated hyaline cylinders within the tubular lumen [12]. Clinical studies also showed renal injuries following supra- or infrarenal abdominal aortic surgery [4, 14, 15].

Mitochondria are key factor involved in skeletal muscle alteration during lower limb IR, and, interestingly, skeletal muscle mitochondrial dysfunctions occur early and are reversible after therapy [16–20]. Accordingly, mitochondria are considered as a central player in cell survival and are main source of ATP and reactive oxygen species generation. Mitochondria are also a central pathway of apoptosis [2].

Protecting remote organ mitochondria might therefore be a goal since mitochondrial dysfunction might lead to the multiorgan failure syndrome. However, very few data [21] are available on the effect of lower limb IR on mitochondrial function of main organ involved in the multiorgan failure syndrome such as the lungs, liver, and kidneys.

The aim of this study was therefore to challenge the hypothesis that lower limb IR results in lung, renal, and/or liver mitochondrial oxidative capacities impairment. We therefore investigated specifically their mitochondrial respiratory chain complexes activities and mitochondrial coupling that might be early markers of remote injuries.

## 2. Methods

### 2.1. Animals

Experiments were performed on 8 to 10 weeks male C57BL6 mice (Depré, France) weighing 20–24 g. Animals were housed in a neutral temperature environment (22 ± 2°C) on a 12 : 12 hour photoperiod and were provided with food and water* ad libitum*. Procedures were conducted in accordance with Helsinki Accords for Humane Treatment of Animals during Experimentation.

### 2.2. Anesthesia, Ischemia-Reperfusion Protocol, and Organ Harvesting

Mice were ventilated with gas mixture of 4% isoflurane (AERRANE, BAXTER S.A.S.) and oxygen in a hermetic anaesthetic induction cage and placed on heating blankets (MINERVE, Esternay, France). Spontaneous ventilation was allowed through an oxygen-delivering mask, with different concentrations of isoflurane depending on the surgical stage (2% during painful stimuli and 1% during latent periods).

Then, a tourniquet was placed around each groin without skin incision. The Sham group (Sham group, *n* = 6) had no ischemia. The ischemia-reperfusion group (IR group, *n* = 6) had a 2-hour ischemia by tightening both tourniquets. To limit potential variability, the same surgeon performed all tourniquet procedures at the same height and strength. Venous and arterial circulation was thus clamped, but no local crush injury was observed.

After a 2-hour reperfusion period, a midline laparotomy was performed, and both kidneys were dissected from the retroperitoneum and harvested by cutting renal vessels and ureters. The liver was then freed, and large specimens were retrieved from both lobes without cutting the hilum to preserve hepatic vessels and continue the thoracic procedure and harvesting.

A midline sternotomy followed. Pleurae were opened, and both lungs were harvested by cutting pulmonary vessels and main bronchi at the level of the hilum. This stage was performed quickly since mice had spontaneous ventilation that was made impossible when pleurae were opened. Mice were then sacrificed by heart harvesting.

### 2.3. Extraction of Lung, Liver, and Kidneys Mitochondria

All operations were carried out on ice. A piece of tissue was placed into buffer A containing 50 mM Tris, 1 mM EGTA, 70 mM Sucrose, and 210 mM Mannitol, pH 7.40 at +4°C. Tissue was finely minced with scissors, placed in buffer A, and homogenized with a tissue grinder. Then, the homogenate was centrifuged at 1300 ×g for 3 min, 4°C. The supernatant was centrifuged at 10,000 ×g for 10 min, 4°C, to sediment mitochondria. Finally, the mitochondrial pellet was washed twice and then suspended in 50 mM Tris, 70 mM Sucrose, and 210 mM Mannitol, pH 7.4 at +4°C. Protein content was routinely quantified with a Bradford assay using bovine serum albumin as a standard.

### 2.4. Study of Mitochondrial Respiratory Chain Complexes Activities and Mitochondrial Coupling

Oxygen consumption was measured polarographically by using a Clark-type electrode (Strathkelvin Instruments, Glasgow, Scotland).

**Figure 1:**
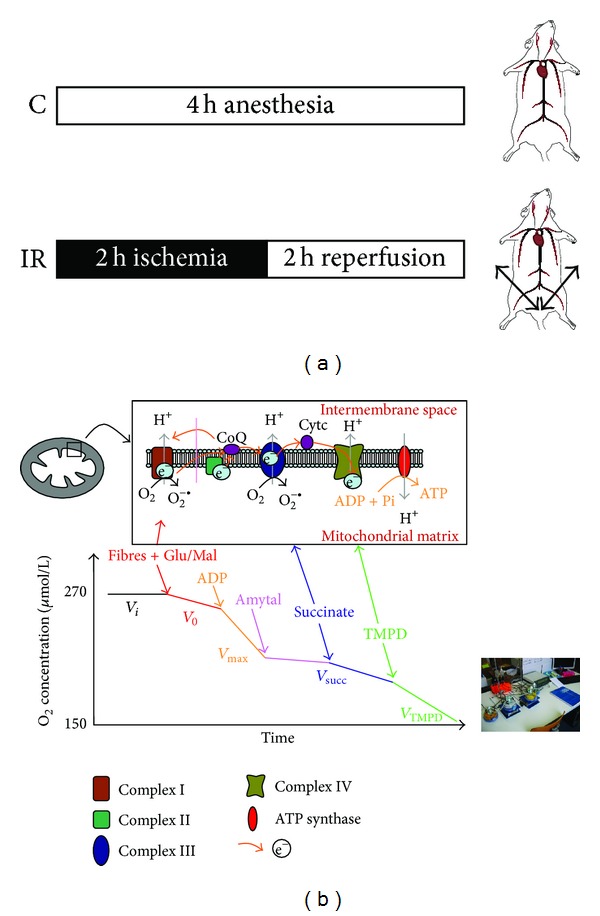
(a) Experimental design. C: control; IR: ischemia-reperfusion. (b) Schematic representation of the mitochondrial respiratory chain with specific substrates and inhibitors. C_I_: complex I (NADH-CoQ reductase), C_II_: complex II (succinate-CoQ reductase), C_III_: complex III (CoQH2-c reductase), C_IV_: complex IV (cytochrome c oxidase, COX), and TMPD: N, N, N′, N′-tetramethyl-p-phenylenediamine dihydrochloride. Schematic oxygraph trace showing oxygen consumption by the permeabilized skeletal myofibers, using indicated substrates and inhibitors: *V*_0_, before ADP; *V*_max⁡_, complexes I, III, and IV activities, using glutamate and malate; *V*_succ_, complexes II, III, and IV activities, using succinate; *V*_TMPD/asc_, complex IV activity using TMPD/Ascorbate.

When maximal fibre respiration (*V*_max⁡_) was recorded, electron flow went through complexes I, III, and IV (Figure 1), because of the presence of glutamate (5 mM) and malate (2 mM). Complex I was blocked with amytal (0.02 mM), and complex II was stimulated with succinate (25 mM). Mitochondrial respiration in these conditions allowed determining complexes II, III, and IV activities (*V*_succ_). After that, N, N, N′, N′-tetramethyl-p-phenylenediamine dihydrochloride (TMPD, 0.5 mM) and Ascorbate (0.5 mM) were added as an artificial electron donor to complex IV. Complex IV activity was then determined as an isolated step of the respiratory chain (*V*_TMPD_).

The degree of coupling between oxidation and phosphorylation was inferred fromthe acceptor control ratio (ACR: *V*_max⁡_/*V*_0_).

### 2.5. Standard Liver and Kidneys Histology

Liver and kidneys tissue sections were stained with haematoxylin-eosin, as previously described [13].

### 2.6. Statistics

SPSS 17.0 for Windows (SPSS Inc., Chicago, IL, USA) was used for statistical analyses. Graphics were generated by GraphPad Prism 4 (GraphPad Software Inc., San Diego, CA, USA). Means were compared between groups using a *t*-test. Results are shown as mean ± standard error (mean ± SEM in graphics). Results with a *P* value less than 0.05 were considered statistically significant.

## 3. Results

Measuring oxygen consumption allowed determining the functional oxidative capacity of each organ mitochondrial function and particularly the relative contribution of the respiratory chain complexes I, II, III, and IV to the global mitochondrial respiratory rate.

### 3.1. Effects of Lower Limb Ischemia-Reperfusion on Lung, Liver, and Kidney Mitochondrial Respiratory Chain Complexes Activities

#### 3.1.1. Lower Limb Ischemia-Reperfusion Impaired Lung Mitochondrial Maximal Oxidative Capacity

**Figure 2:**
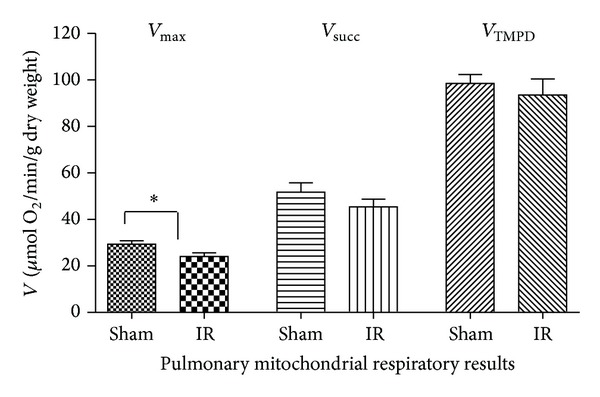
Effects of lower limb ischemia/reperfusion on lung mitochondrial respiratory chain complexes activities. (A) *V*_max⁡_, complexes I, III, and IV activities, using glutamate and malate; (B) *V*_succ_, complexes II, III, and IV activities, using succinate; (C) *V*_TMPD/asc_, complex IV activity using TMPD/Ascorbate, as mitochondrial substrates. Data are presented in Sham and after ischemia-reperfusion (IR). Results are expressed as means ± SEM; *P < 0.05.

IR decreased lung maximal oxidative capacity (Figure 2). Thus, *V*_max⁡_ decreased from 29.4 ± 3.3 to 24.0 ± 3.7 *μ*mol O_2_/min/g dry weight (−18.4%, *P* = 0.042; Figure 1). *V*_succ_ decrease was not statistically significant after IR (51.8 ± 8.9 versus 45.4 ± 7.5 *μ*mol O_2_/min/g dry weight; −12.4%, *P* = 0.254), and *V*_TMPD_ was nearly identical in both groups (98.6 ± 8.3 versus 93.6 ± 15.4 *μ*mol O_2_/min/g dry weight; −5.1%, *P* = 0.540).

Thus, as inferred from the data obtained with each specific substrate we observed that complex I of the lung mitochondrial respiratory chain was impaired by lower limb IR.

#### 3.1.2. Lower Limb Ischemia-Reperfusion Did Not Alter Liver Mitochondrial Function

**Figure 3:**
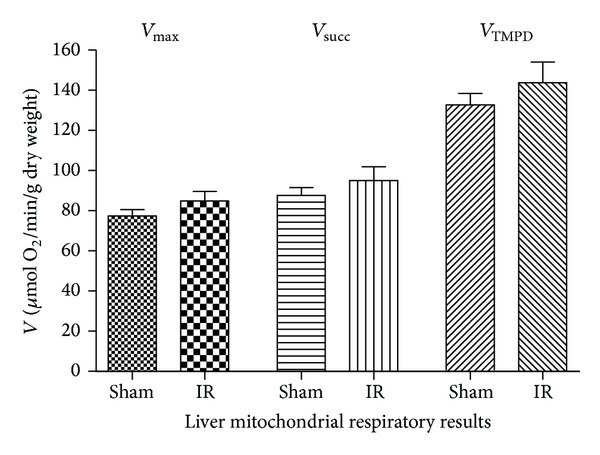
Effects of lower limb ischemia/reperfusion on liver mitochondrial respiratory chain complexes activities. (A) *V*_max⁡_, complexes I, III, and IV activities, using glutamate and malate. (B) *V*_succ_, complexes II, III, and IV activities, using succinate. (C) *V*_TMPD/asc_, complex IV activity using TMPD/Ascorbate, as mitochondrial substrates. Data are presented in Sham and after ischemia-reperfusion (IR). Results are expressed as means ± SEM.

Indeed, lower limb IR caused a slight, not statistically significant, increase in liver mitochondrial respiratory values (Figure 3). *V*_max⁡_ increased from 77.4 ± 7.7 to 84.8 ± 12.0 *μ*mol O_2_/min/g dry weight (+9.6%, *P* = 0.234; Figure 2). *V*_succ_ increased from 87.7 ± 9.4 to 95.1 ± 16.7 *μ*mol O_2_/min/g dry weight (+8.4%, *P* = 0.367), and *V*_TMPD_ increased from 132.8 ± 13.9 to 143.7 ± 25.3 *μ*mol O_2_/min/g dry weight (+8.2%, *P* = 0.373).

Thus, lower limb IR failed to alter liver mitochondrial function, and all complexes of the mitochondrial respiratory chain (complexes I, II, III, and IV) showed a similar 9% trend to increase their activities.

#### 3.1.3. Lower Limb Ischemia-Reperfusion Enhanced Renal Mitochondrial Function

**Figure 4:**
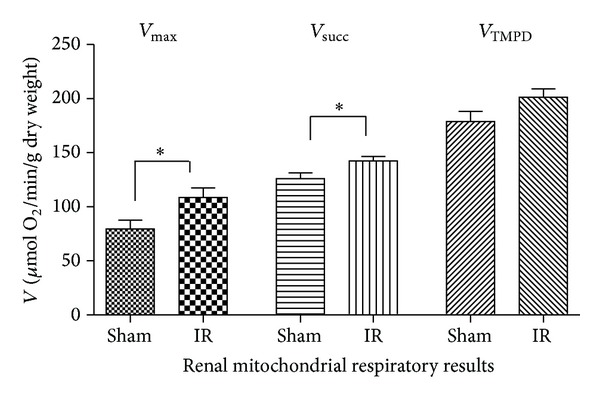
Effects of lower limb ischemia/reperfusion on kidney mitochondrial respiratory chain complexes activities. (A) *V*_max⁡_, complexes I, III, and IV activities, using glutamate and malate. (B) *V*_succ_, complexes II, III, and IV activities, using succinate. (C) *V*_TMPD/asc_, complex IV activity using TMPD/Ascorbate, as mitochondrial substrates. Data are presented in Sham and after ischemia-reperfusion (IR). Results are expressed as means ± SEM; *P < 0.05.

Both renal *V*_max⁡_ and *V*_succ_ were significantly increased after IR (Figure 4). *V*_max⁡_ increased from 79.5 ± 19.9 to 108.6 ± 21.4 *μ*mol O_2_/min/g dry weight (+36.6%, *P* = 0.035; Figure 3). *V*_succ_ increased from 126.0 ± 13.4 to 142.4 ± 10.4 *μ*mol* *O_2_/min/g dry weight (+13.0%, *P* = 0.039). *V*_TMPD_ tended to increase after IR, but this failed to reach statistical significance (178.9 ± 22.8 versus 201.3 ± 19.1 *μ*mol O_2_/min/g dry weight; +12.5%, *P* = 0.094).

Thus, complexes I, II, and III activities of the mitochondrial renal respiratory chain were enhanced after IR.

### 3.2. Effects of Lower Limb Ischemia-Reperfusion on Lung, Liver, and Kidney Mitochondrial Coupling

**Figure 5:**
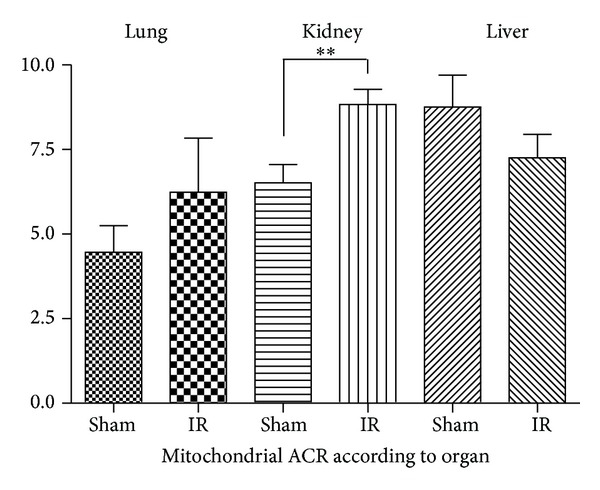
Effects of lower limb ischemia/reperfusion on lung, liver, and kidney mitochondrial coupling. ACR: acceptor complex ration (*V*_max⁡_/*V*_0_). Data are presented in Sham and after ischemia-reperfusion (IR). Results are expressed as means ± SEM; *P < 0.05.

Mitochondrial coupling was enhanced in kidney after lower limb ischemia-reperfusion (6.5 ± 1.3 versus 8.8 ± 1.1; *P* = 0.008 in Sham and IR mice, resp.). No significant differences were observed concerning, respectively, the lung (4.5 ± 1.8 versus 6.2 ± 3.6, *P* = 0.346) and the liver (8.8 ± 2.3 versus 7.3 ± 1.7, *P* = 0.229), in Sham and IR, respectively (Figure 5).

### 3.3. Effects of Lower Limb Ischemia-Reperfusion on Liver and Kidney Structure

Liver and kidneys tissue sections, stained with haematoxylin-eosin, demonstrated normal architecture and thus no change was observed after IR (data not shown).

## 4. Discussion

The main results of this study are to demonstrate for the first time that tourniquet-induced bilateral hindlimb ischemia-reperfusion (1) impairs lung maximal oxidative capacity, (2) does not modify liver mitochondrial function, and (3) unexpectedly stimulates kidney mitochondrial respiratory chain complexes activities and coupling in experimental mice.

Lower limb ischemia-reperfusion procedures are routinely performed throughout the world using either artery clamping or tourniquet. Particularly, tourniquet is widely used during orthopaedic surgeries in order to reduce bleeding in the operating zone. These procedures are associated not only with local skeletal muscle mitochondrial dysfunctions but also with remote lesions, generally assessed histologically or at a global, functional level. Since remote lesions largely participate in patient's morbidity and mortality, a better knowledge of pathophysiological mechanisms involved might be useful to open new therapeutic perspectives. In this view, investigation on remote organ mitochondrial functions might be particularly interesting since mitochondrial dysfunction occurs early in skeletal muscles submitted to IR and is likely to be avoided using adapted strategies of ischemic or pharmacologic conditioning [22–24].

Although we showed no lung mitochondrial alteration after lower limb IR secondary to aortic cross-clamping in rats [21], the present study demonstrates the implication of mitochondria in remote pulmonary lesions after tourniquet-induced bilateral lower limb ischemia-reperfusion in mice. Accordingly, pulmonary injuries occur after major vascular surgeries and are largely involved in patient's mortality and morbidity [6–12]. Lungs are privileged sites for remote lesions after skeletal muscle ischemia-reperfusion, mostly by attracting and sequestrating leukocytes [9, 11]. Raijmakers et al. showed enhanced protein leak index in lung parenchyma after aortic surgery [25], and Adembri et al. found increased need in oxygen delivery to maintain normal systemic oxygenation, an indirect proof of alveolar alteration [6]. Interestingly, an experimental model of lung transplantation characterized by pulmonary ischemia-reperfusion also resulted in mitochondrial dysfunction likely leading to energy loss, oxidative stress, apoptosis, and ultimately to organ failure [26]. Our data are in line with these results, although mitochondria were not deprived of oxygen or nutrients.

Taken together, these data support that circulating factors might be responsible for the reduced mitochondrial respiratory chain complexes observed after lower limb IR. On the other hand, incomplete hypoxia during aortic cross-clamping might be associated with a reduction of the deleterious factors released, as compared to tourniquet-induced complete bilateral hindlimb ischemia [21]. Alternatively, mice might be more prone to IR damage than rats [27], possibly suggesting species differences and supporting specific studies in humans.

The circulating factors involved in remote organ damage are not totally known, but reactive oxygen species, cytokines, and complements factors associated with increased circulating leukocytes are likely to play a key role. Accordingly, studies reducing these factors allow organ protection, including lung protection [28–30]. Stimulation of the protective pathways such as RISK, SAFE, and heat shock protein might also be useful [2, 28, 30].

Liver mitochondrial function was not impaired in our study. This is consistent with the fact that humans liver dysfunction is observed mainly when lower limb IR is complicated by a shock, linked to multiorgan failure [13], and supports a greater resistance of the liver. Accordingly, no histological change was observed in the liver in this study, and increased hepatic enzyme after experimental lower limb IR has been attributed to muscle rather than to liver dysfunction [21].

Concerning the kidneys, we expected to observe impairments in mitochondrial respiration. Indeed, many experimental and clinical reports demonstrated that kidneys are key targets in the setting of IR. Thus, Miller et al. demonstrated a “relationship between intraoperative leg ischemia and postoperative renal failure, providing epidemiological evidence that the ischemic leg may be an important contributor to rhabdomyolysis-like renal morbidity after thoracoabdominal aortic surgery” [15]. The importance of peripheral muscle ischemia-reperfusion and rhabdomyolysis as a significant factor in the renal alterations was also stressed in other studies [4, 31]. Tallgren et al. noticed increased preoperative albuminuria and postoperative acute renal dysfunction in 22% of patients with a 4% incidence of postoperative renal failure [14]. They incriminated changes in renal perfusion (hypotension, low cardiac output), nephrotoxic agents, inflammatory and neuroendocrine stress, and rhabdomyolysis. Interestingly, Ali et al. demonstrated that renal impairment well responded to therapeutic approach such as ischemic preconditioning [5].

The kidney functional enhancement observed in this study might correspond to the fact that kidneys had to filter circulating toxic whose numbers highly increase during lower limb IR. Therefore, their energetic capacities needed to be improved. A secondary damage might then occur later, when kidneys mitochondrial defences are overloaded by a higher amount of oxidative stress and inflammatory factors. Accordingly, we did not observe histological change in the kidney early after reperfusion, but other authors demonstrated tubular cell necrosis after 24 h of reperfusion following a 2-hour femoral artery occlusion [32].

*Limitations of the Study*. There is a possibility that damaged mitochondria drop out from the supernatant during the isolation procedure but it is unlikely a major event since studies demonstrated decrease in oxidative capacities after centrifugation, supporting the presence of impaired mitochondria [13, 33].

A temporal response to ischemia looking at different time point of reperfusion might be very interesting. This might help discover new pattern of mitochondrial activities in different organs, and, particularly, this might allow determining when and why lung and kidneys functions are impaired after lower limb IR. Further, this will pave the way for new therapeutic approaches. In this view, circulating leukocytes and platelets or released molecules might be analysed by performing cellular depletion or serum transfusion experiments and proteomic assessment of the plasma.

## 5. Conclusions

Tourniquet-induced bilateral hindlimb ischemia-reperfusion leads to differential remote organ mitochondrial effects. Liver appeared to be well preserved but lungs were early damaged. Better knowing the pathophysiology of lung injury might help to focus therapeutic approach on lung mitochondrial functions, since they were beneficial for skeletal muscle in lower limb IR settings. Additionally, further kinetic studies will be useful to confirm whether and when kidneys mitochondrial dysfunctions occur and how to reduce these remote organ mitochondrial dysfunctions.
